# Longitudinal assessment of the impact of COVID-19 infection on mask-wearing behaviors

**DOI:** 10.1186/s12889-024-19776-0

**Published:** 2024-08-16

**Authors:** Danielle Pham, Angel Lomeli, Nicole H. Goldhaber, Holly D. Valentine, Rob Knight, Christopher A. Longhurst, Louise C. Laurent, Marni B. Jacobs

**Affiliations:** 1 University of California, San Diego Herbert Wertheim School of Public Health and Human Longevity Science, La Jolla, USA; 2 San Diego School of Medicine, Department of Obstetrics, Gynecology, and Reproductive Sciences, Division of Maternal Fetal Medicine, University of California, 9300 Campus Point Dr., MC 7433, , La Jolla, USA; 3 San Diego School of Medicine, Department of Surgery, University of California, La Jolla, USA; 4 San Diego EXCITE Laboratory, University of California, La Jolla, USA; 5 San Diego School of Medicine, Department of Pediatrics, University of California, La Jolla, USA; 6 Department of Computer Science and Engineering, University of California, San Diego Jacobs School of Engineering, La Jolla, USA; 7 San Diego School of Medicine, Department of Medicine, University of California, La Jolla, USA

**Keywords:** COVID-19, Mask use, Risk perception, University-affiliated individuals, Infection status

## Abstract

**Background:**

Wearing a mask was a crucial component in slowing the COVID-19 pandemic. However, little is known about the intersectionality between mask usage, risk perception, and infection. The purpose of this study was to investigate whether risk perceptions and masking behaviors are associated with contracting SARS-CoV-2 and how contracting SARS-CoV-2 subsequently changes masking behaviors in specific situations.

**Methods:**

This cohort study utilized survey data from the UC San Diego ZAP COVID-19 study (*n* = 1,230) to evaluate the risk of contracting SARS-CoV-2 in relation to baseline risk perceptions and masking behaviors in various situations and how contracting SARS-CoV-2 affects subsequent masking behavior.

**Results:**

We found that more consistent self-reported mask use in indoor public spaces (*p* = 0.03) and in other people’s houses (*p* = 0.002) was associated with remaining free of SARS-CoV-2 infection. We also found that contracting SARS-CoV-2 was associated with a subsequent increase in mask use in other people’s houses (*p* = 0.01).

**Conclusions:**

Our findings suggest that consistent mask use is correlated with decreased infection and that contracting SARS-CoV-2 may modify mask use behaviors in high-risk situations. These findings may help inform future public health messaging for infectious disease prevention.

**Trial registration:**

This study has not been previously registered as it is an observational study. There was no pre-registration of the analytic plan for the present study.

**Supplementary Information:**

The online version contains supplementary material available at 10.1186/s12889-024-19776-0.

## Background

The COVID-19 pandemic has been one of the most significant global crises in recent history, affecting virtually every aspect of human life. First identified in late 2019, COVID-19 is a highly contagious respiratory illness caused by the novel coronavirus SARS-CoV-2. It spreads primarily through airborne respiratory droplets and can also be transmitted by touching surfaces contaminated with the virus and then touching one’s eyes, nose, or mouth [1]. Persons infected with COVID-19 exhibit a wide range of symptoms, including fever, cough, shortness of breath, fatigue, loss of taste or smell, and muscle pain [2]. In severe cases, COVID-19 infection can lead to pneumonia, acute respiratory distress syndrome, organ failure, and even death [2]. However, certain individuals remain asymptomatic after contracting COVID-19, yet they may still exhibit high levels of infectiousness [3]. There have been more than 104 million confirmed cases of COVID-19 in the United States since April 12, 2021, with a reported 1,127,928 total deaths associated with this disease [4]. Moreover, discernible patterns emerge that highlight certain populations bearing a disproportionate burden of its impact. Notably, individuals who are elderly, are racial and ethnic minorities, reside in low-income communities, and are affected by underlying health conditions have heightened vulnerability, particularly to severe adverse outcomes [5, 6].

Wearing masks has been proposed as a crucial measure for mitigating the spread of COVID-19 and safeguarding public health. Masks play a vital role in mitigating the spread of COVID-19 by effectively preventing viral-laden fluid droplets from infected persons from transforming into aerosols that can remain in the air for hours [7, 8]. Notably, certain mask types, such as the N95 and KN95 respirators, can also prevent aerosols from being inhaled by uninfected individuals [9]. The fundamental objective underlying mask usage revolves around reducing COVID-19 symptom severity, which tends to worsen when the virus infiltrates the lower respiratory tract [10]. By decreasing the volume of viral particles that individuals respire, masks play an instrumental role in diminishing the likelihood of viral infiltration into the lower airways, hence mitigating the potential severity of the disease [11]. Despite the reported benefits of wearing a mask, opposition persists toward this preventive practice. Some commonly cited reasons for opposition to wearing masks include a perceived lack of effectiveness, physical discomfort, and being perceived as “inappropriate” [12, 13].

The majority of existing research on risk mitigation and COVID-19 pertains to how risk perceptions and self-perceptions influence positive health behaviors such as vaccine uptake, social distancing, and wearing a mask [12, 14–17]. However, less is known about the influence of contracting COVID-19 on risk perception and protective health behaviors. While a previous study did not find any correlation between previous COVID-19 infection and physical distancing, mask wearing, and hand hygiene [18], more studies are necessary to fully understand how contracting SARS-CoV-2 affects individual risk perception or health behaviors in different situations. Research in other areas, such as cancer and chronic diseases, suggests that disease diagnosis can positively influence health behaviors [19, 20]. However, whether this phenomenon translates to temporary illnesses such as infectious diseases is unclear, although understanding this association may help guide public health strategies. Here, we use data from a longitudinal study of self-reported masking behaviors and COVID-19 infections to examine whether an individual’s masking behavior is associated with contracting COVID-19 and whether contracting COVID-19 is associated with changes in risk perception and masking behaviors.

## Methods

*Study Design:* Data for the present study were drawn from a prospective cohort study designed to evaluate SARS-CoV-2 antibody levels and factors associated with contracting SARS-CoV-2 (Neutralizing Antibody Project for COVID-19, ZAP COVID-19). Individuals 18 years and older affiliated with the University of California, San Diego (UCSD) and UCSD Health were eligible to participate. Participants were recruited in person at onsite events and through email invitations, mailing lists, and UCSD newsletters. All study procedures were approved by the Institutional Review Board at the University of California, San Diego; all participants provided electronic written informed consent prior to participation. Study methodology and results are reported following the Strengthening the Reporting of Observational Studies in Epidemiology (STROBE) Statement for cohort studies [21].

### Measures

As part of the study, participants were asked to complete surveys regarding COVID-19 risk perceptions and masking behaviors at enrollment (baseline) and at 30 and 90 days post-enrollment. Demographic information and medical comorbidities were also collected at the time of study enrollment. At UCSD, both employee health and student health use a shared instance of a vendor-supplied electronic health record (EHR) system (Epic Systems, Verona, WI) [22, 23]. This system ensures that electronic medical records are automatically created for all UCSD students and staff at the time of affiliation to track required vaccinations and testing, as well as to enable any visits to student or employee health. All the surveys were completed electronically in the EHR as part of the e-Check-In process (prior to the first visit) or via EHR messaging with a linked survey at specified intervals following the initial study visit. Risk perceptions were assessed using the question “What do you think your risk is of contracting COVID-19?”, with responses given on a scale of 1 to 7, with 1 indicating a very low risk and 7 indicating a very high risk. For the purpose of analysis, the scale was condensed to “low risk” (responses 1–3), “average risk” (4), and “high risk” (5–7). Sensitivity analyses were conducted using an alternate classification of the scale expanding the middle (“average”) category with similar results obtained. Masking behaviors were assessed across four distinct scenarios, including outdoor situations when individuals were within 6 feet of others; indoor public settings; public transportation (e.g., bus, train, or rideshare); and indoor social gatherings at a friend or family member’s residence with whom the participant did not share living quarters. Participants were asked to respond to the question “During the last 2 weeks when you left your house, how often have you worn a mask that covers your mouth and nose in the following situations?”, which was rated on a scale ranging from “never”, to “rarely”, “sometimes”, “usually”, and “always”. “Not applicable” was also an allowable response if the participant had not been in the specified situation. For analysis, “not applicable” was excluded, “never” and “rarely” were condensed, and “usually” and “always” were condensed. Information on demographics, lifestyle factors, and COVID-19 risk factors obtained from the initial survey was used to record age, sex, ethnicity/race, vaccine status, current medications, and medical comorbidities. Height and weight were self-reported.

Information on SARS-CoV-2 infections was obtained from the EHR. Participants were considered to have had a SARS-CoV-2 infection if they tested positive on a SARS-CoV-2 PCR test. During this period, UCSD provided all students and employees with no-cost, easy-to-access testing for SARS-CoV-2, with results recorded in the EHR. Prior to October 2022, unvaccinated students and employees were required to request a formal vaccine exemption and to test twice per week, while vaccinated students and employees were encouraged to test weekly or at any time they experienced symptoms. The study population was highly vaccinated (98.7%), and testing rates were high during the study period (median number of tests = 8).

### Study population

**Fig. 1:**
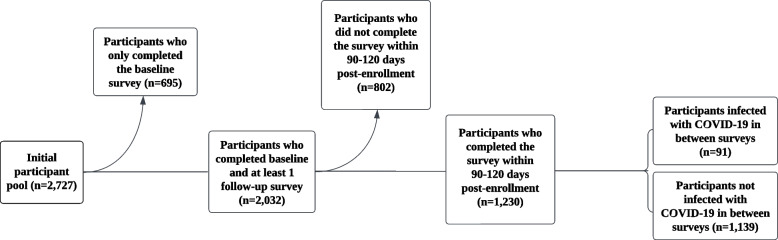
Participant exclusion and inclusion flowchart

The initial participant pool for this study consisted of 2,727 individuals who consented to participate in the study from January to December 2022. For the purposes of the present study, the 90-day survey time point was selected as the time point of interest to allow time for both infections and possible behavioral changes to occur. We excluded 695 participants who completed only the baseline questionnaire and lacked the necessary follow-up data for analysis; we further excluded 802 participants who did not complete the survey within the specified timeframe of 90–120 days post enrollment to mitigate the confounding influence of time (Fig. 1). Thus, the final analytic sample included 1,230 participants. We divided participants into two groups for analysis, those with no documented COVID-19 infection and those with COVID-19 infection; these groups included individuals who contracted COVID-19 between the first (enrollment) and second survey time points (90–120 days). Compared to those who were excluded due to insufficient follow-up, those included in the analysis were slightly more likely to be female (69.8% vs. 66.3%, *p* = 0.05) or older (40.4 years vs. 36.5, *p* < 0.001); to have Hispanic/Latinx/Spanish origin (25.2% vs. 2.3%, *p* < 0.0001); and to be less likely to be non-Hispanic White (41.9% vs. 46.5%, *p* = 0.02), Asian (23.6% vs. 27.2%, *p* = 0.03), or Other/Mixed (4.5% vs. 14.2%, *p* < 0.0001) and to have an unknown race/ethnicity (1.2% vs. 5.4%, *p* < 0.0001).

### Analysis

Demographics, baseline risk perceptions, and mask-wearing behaviors were compared between the infected and non-infected groups using the chi-square test for each category individually compared to all others or t test, as appropriate, to evaluate specific associations. The demographic data of the participants not included in the analysis were also compared with the demographic data of the participants included in the analysis to assess possible bias in the included and excluded samples. To facilitate analysis of behavioral change, ordinal scales for risk perception and masking behaviors were transformed into numerical scales ranging from 1 (“very low risk”) to 7 (“very high risk”) for risk perception and 1 (“never”) to 5 (“always”) for masking in specified situations. Subsequently, the difference between the most recent survey score (survey between days 90 and 120) and the baseline survey score (day 0) was computed for each participant. Negative differences indicated a decrease in risk perception or masking behaviors, while positive differences denoted an increase. These differences were then categorized into three groups: decreased risk perception/masking behaviors (negative difference), no change in risk perception/masking behaviors (difference = 0) and increase in risk perception/masking behaviors (positive difference). These differences were also assessed as continuous incremental changes. A chi-square test for categorized outcome change and a t-test for continuous outcome calculations were used to ascertain whether changes in risk perception and masking behavior from baseline to follow-up differed between the infected and non-infected groups. Significant differences in categorical outcomes were further explored using odds ratios (ORs), which were used to assess the association between contracting COVID-19 during the survey period and changes in risk behaviors, with the “no change” group serving as the reference group, evaluated as separate dichotomous ratios. A *p* value < 0.05 was considered to indicate statistical significance in all analyses. All analyses were conducted using RStudio 2022.02.0.

## Results

The characteristics of the study sample are shown in Table 1. No differences in sex, race or ethnicity were noted across infection groups; however, infected participants were slightly younger (mean = 37.4) than non-infected participants were (mean = 40.7, *p* = 0.02). The ages of the participants ranged from 18 to 80 years. Slight differences in mask use according to demographic variables were noted at baseline; participants identified as Asian or black were more likely to report consistent mask use in various situations (see Supplemental Table 1).

**Table 1:** Demographics

	**Infected, n (%)**	**Not Infected, n (%)**	***P*-Value**
**Sex**			
Male	29 (31.9%)	338 (29.7%)	0.66
Female	62 (68.1%)	797 (70.0%)	0.70
Unknown	0 (0.0%)	4 (0.3%)	0.60
**Race/Ethnicity**			
Non-Hispanic White	40 (44.0%)	475 (41.7%)	0.67
Hispanic/Latino/Spanish Origin	23 (25.3%)	287 (25.2%)	0.98
Asian American	17 (18.7%)	273 (24.0%)	0.25
Black/African American	1 (1.1%)	25 (2.2%)	0.48
American Indian or Alaskan Native	0 (0.0%)	7 (0.6%)	0.46
Native Hawaiian or Pacific Islander	2 (2.2%)	11 (1.0%)	0.29
Other or Mixed	6 (6.6%)	49 (4.3%)	0.33
Unknown	2 (2.2%)	12 (1.0%)	0.29
**Age [Mean (SD)]**	37.4 (10.6)	40.7 (14.1)	0.02^*^
**Previously Infected**	13 (14.3%)	90 (7.9%)	0.06
**Completed Vaccine Schedule with Three Doses**	85 (93.4%)	1054 (92.5%)	0.95

*P*-value is associated with infection status

^*^p < 0.05; ^**^p < 0.01; ^***^p < 0.001

^a^Yes: Usually, Always, Sometimes at baseline for wearing a mask outdoors and less than 6 feet away from another person

Associations between baseline risk perception and mask use and subsequent SARS-CoV-2 infection incidence are shown in Table 2. There was no difference in baseline risk perception or masking behaviors in outdoor spaces when individuals were less than 6 feet away or when they were on public transport between the infection groups. However, the group not infected during follow-up was more likely than the infected group to report usually or always wearing a mask in indoor public spaces (86.2% vs. 77.8%, *p* = 0.03) or in other people’s houses (23.2% vs. 8.5%, *p* = 0.002), while the infected group was more likely to report only sometimes masking (16.7% vs. 7.7%, *p* = 0.003) in indoor public spaces. A trend towards never or rarely masking other people’s homes was also noted in the infected group (68.3% vs. 57.3%, *p* = 0.05).

**Table 2:** Baseline risk perceptions and mask use in different situations by infection group

	Infected n (%)	Not Infected n (%)	Difference in %	*P*-Value
**Risk Perception**				
Low Risk	34 (37.4%)	514 (45.9%)	8.5	0.12
Average Risk	43 (47.3%)	420 (37.5%)	9.8	0.06
High Risk	14 (15.3%)	185 (16.6%)	1.3	0.75
**Masking Behavior in Outdoor Spaces When Less Than 6 Feet Away**				
Never/Rarely	17 (18.9%)	214 (18.9%)	0.0	1.00
Sometimes	21 (23.3%)	181 (16.1%)	7.2	0.08
Usually/Always	52 (57.8%)	733 (65.0%)	7.2	0.17
**Masking Behavior in Indoor Public Spaces**				
Never/Rarely	5 (5.5%)	70 (6.1%)	0.6	0.82
Sometimes	15 (16.7%)	87 (7.7%)	9.0	0.003^***^
Usually/Always	70 (77.8%)	976 (86.2%)	8.4	0.03^*^
Masking Behavior in Public Transport				
Never/Rarely	3 (6.1%)	33 (5.7%)	0.4	0.91
Sometimes	2 (4.1%)	26 (4.5%)	0.4	0.90
Usually/Always	44 (89.8%)	522 (89.8%)	0.0	1.00
Masking Behavior in Other People’s Houses				
Never/Rarely	56 (68.3%)	547 (57.3%)	11.0	0.05
Sometimes	11 (13.4%)	184 (19.5%)	6.1	0.18
Usually/Always	15 (8.5%)	214 (23.2%)	14.7	0.002^**^

^*^p < 0.05; ^**^p < 0.01; ^***^p < 0.001

Table 3 displays the changes in risk perception and masking behaviors from baseline to follow-up in different situations for both the infected and non-infected groups. There was no significant difference in the changes in risk perception (*p* = 0.78) or in masking behaviors in outdoor spaces when less than 6 feet were removed (0.23), in indoor public spaces (*p* = 0.25), or in public transportation (*p* = 0.81). However, a significant difference in masking behavior while in other people’s houses was noted (*p* = 0.01), with the infected group having 2.43 times greater odds of increasing their mask use than the non-infected group (95% CI = 1.24–4.74) compared to no change in mask use between the first time point and the second time point.

**Table 3:** Changes in risk perception and masking behaviors over time between infected and non-infected groups

	**Infected, n (%)**	**Not Infected, n (%)**	***P*-Value**	**OR**	**CI**
**Risk Perception**			0.78		
No Change	44 (55.7%)	576 (53.5%)			
Decreased	12 (15.2%)	198 (18.4%)			
Increased	23 (29.1%)	303 (28.1%)			
Total	79	1077			
**Masking Behavior in Outdoor Spaces When Less Than 6 Feet Away**			0.23		
No Change	33 (37.5%)	522 (46.9%)			
Decreased	50 (56.8%)	543 (48.7%)			
Increased	5 (5.7%)	49 (4.4%)			
Total	88	1114			
**Masking Behavior in Indoor Public Spaces**			0.25		
No Change	52 (59.1%)	652 (57.8%)			
Decreased	30 (34.1%)	437 (38.7%)			
Increased	6 (6.8%)	40 (3.5%)			
Total	88	1129			
**Masking Behavior in Public Transport**			0.81		
No Change	28 (71.8%)	321 (71.8%)			
Decreased	10 (25.6%)	105 (23.5%)			
Increased	1 (2.6%)	21 (4.7%)			
Total	39	447			
**Masking Behavior in Other People’s Houses**			0.01^*^		
No Change	43 (58.9%)	554 (62.4%)	reference	reference	–
Decreased	17 (23.3%)	264 (29.8%)	0.53	0.83	[0.46, 1.48]
Increased	13 (17.8%)	69 (7.8%)	0.009	2.43	[1.24, 4.74]

^*^p < 0.05; ^**^p < 0.01; ^***^p < 0.001

Table 4 presents the average changes in risk perception and mask-wearing behaviors with the mean change reported based on calculated changes from the baseline to the second survey. Notably, risk perception increased in both groups from baseline to follow-up, while masking in outdoor spaces when less than 6 feet were removed exhibited the greatest decrease in masking behaviors for both the infected and non-infected groups. While no significant difference in absolute change in behaviors was noted between groups, participants not infected during follow-up showed a trend towards greater decreases in masking behavior in indoor public spaces (-0.49 vs. -0.33, *p* = 0.07) or masking in other people’s houses (-0.30 vs. -0.13, *p* = 0.06) compared to infected participants.

**Table 4:** Change in risk perception and mask wearing behaviors based on SARS-CoV-2 infection during follow-up

	**Infected, mean [SD]**	**Not Infected, mean [SD]**	***P*-Value**
Risk Perception	0.18 [0.76]	0.12 [0.83]	0.50
Masking Behavior in Outdoor Spaces When Less Than 6 Feet Away	-0.80 [0.92]	-0.68 [0.91]	0.23
Masking Behavior in Indoor Public Spaces	-0.33 [0.69]	-0.49 [0.82]	0.07
Masking Behavior in Public Transport	-0.32 [0.70]	-0.28 [0.79]	0.64
Masking Behavior in Other People’s Houses	-0.13 [0.93]	-0.30 [0.81]	0.06

## Discussion

In this study, we aimed to investigate the impact of different masking behaviors on contracting COVID-19, as well as the influence of contracting SARS-CoV-2 on risk perception and masking behaviors. We found that more consistent usage of masks in indoor public spaces and in other people’s homes was associated with remaining free of SARS-CoV-2 infection for 90 to 120 days following self-reported masking behaviors. These findings align with other studies, which have shown associations between increased mask use and a reduced risk of contracting COVID-19, as outlined in a review by Brooks and Butler [14]. This can be explained by the use of masks, which primarily function by preventing the virus from spreading from infected individuals while also exhibiting some effectiveness in reducing the inhalation of the virus by non-infected individuals. Interestingly, despite these differences in mask wearing behaviors, there was no significant difference in baseline risk perception between groups, although studies have reported associations between risk perception and protective health behaviors [24, 25]. This may be explained by the different mask mandates in public areas and masking preferences in different people’s homes that the participants had visited, which may have influenced each individual’s masking behaviors.

Overall, our findings indicate a trend towards decreased mask use in both groups, despite an overall increase in risk perception. Additionally, when examining the impact of contracting COVID-19 on masking behaviors, we found that those who were infected with SARS-CoV-2 during follow-up were more likely to report increasing their mask use when in other people’s homes, though not in any other situations explored. This finding may be related to a fear of infecting others knowing they had been infected or an individual’s fear of reinfection following exposure to COVID-19 [26], although some studies have reported a decrease in risk mitigation behaviors following COVID-19 infection due to the perception that individuals acquired long-term immunity [27]. Likewise, in both indoor public spaces and other people’s houses, the group of infected individuals exhibited a smaller mean reduction in masking behavior, though this trend could be attributed to the lower baseline values observed in this group.

The strengths of our study include its large sample size, prospective design, and concrete assessment of SARS-CoV-2 infection, which enhances the reliability of our findings. We were able to establish a relationship between contracting COVID-19 and its impact on masking behaviors due to the longitudinal nature of our cohort study. Additionally, we assessed multiple masking scenarios rather than general masking overall to evaluate specific scenarios in which masking behaviors and risk perceptions may differ.

It is important to acknowledge and address several limitations inherent in our study. First, we experienced attrition throughout the course of our study and noticed differences in the proportions of males and females; individuals who were Hispanic/Latino/Spanish, Other/Mixed, or Unknown; and in the average age between the sample we analyzed and the sample that was not analyzed, potentially introducing bias and affecting the representativeness of our data. Notably, individuals who self-identified as Hispanic/Latino/Spanish origin accounted for 25.2% of our analyzed population but constituted only 2.3% of the excluded group. These findings challenge previous reports suggesting that individuals identifying as Hispanic were less inclined to participate in COVID-19 testing [28] and allowed us to include an assessment of a frequently understudied population. This high participation rate may reflect the large proportion of the overall UCSD population identified as Hispanic/Latino (27.5%), potentially increasing comfort in testing and research participation. More research should be conducted to determine the nature of the differences in testing and research participation in different populations. Additionally, the interpretation of our survey responses, which employed categories such as “never, rarely, sometimes, usually, and always”, may have varied among participants, potentially introducing subjectivity and imprecision into our analysis. A specific limitation within this design pertains to instances where individuals responded “not applicable” to various scenarios. In such cases, it remains unclear why these individuals considered the situations not applicable, which might include the possibility that a “not applicable” response may signify a preference for avoidance of risky situations over mask-wearing. We also recognize the potential influence of unmeasurable confounding factors, such as the cessation or loosening of mask mandates during the study period or “pandemic fatigue”, characterized by feelings of exhaustion and irritation resulting from prolonged mask wearing [29]. Furthermore, approximately 70% of our participants were enrolled within the first two months of the study, making our results significantly influenced by the peak of the Omicron wave [30]. It is likely that multiple factors influence masking behaviors, and behaviors may be mediated by other intervening variables such as social distancing. Thus, these external influences and other correlated behaviors could have impacted participants’ behaviors and may have affected the associations observed in our study. Finally, our study involved only individuals associated with a large academic institution, which limits the generalizability of our findings, and we were unable to account for whether participants were students, staff, or faculty members, which may have influenced risk perceptions and masking attitudes due to potential variations in age and experience. Nonetheless, this study highlights the importance of masking to prevent the spread of infection even in a highly vaccinated population with access to testing. Future research is needed to investigate the impact of contracting COVID-19 on mask-wearing behaviors in different populations and evaluate the effectiveness of interventions aimed at increasing mask use. Qualitative research could provide deeper insights into the complex factors influencing individuals’ attitudes and behaviors regarding mask wearing.

Overall, our study contributes to the expanding body of research on mask-wearing during the COVID-19 pandemic. Whereas existing studies have delved into the influence of risk perceptions and masking behaviors in contracting COVID-19, the reverse relationship has not yet been fully discussed. Although we do not believe that a special intervention would have a specific benefit in the context of individuals who have previously been infected with COVID-19, studies such as the present one that look into this reverse relationship to better understand the influence of contracting COVID-19 on risk perceptions and health behaviors may still help inform public masking policies and campaigns. Within the context of emerging and circulating infectious diseases, identifying populations at high risk for noncompliance with policies such as mask mandates is increasingly important, and targeted interventions using theories such as the Health Belief Model should be created [31]. It is important that public health advocates work closely with community leaders to identify potential barriers when creating interventions to ensure that prevention strategies have the highest possible impact on community health and well-being.

### Supplementary Information

Supplementary Material 1

## Data Availability

The datasets generated and/or analyzed during the current study are not publicly available due to due to privacy laws related to private health information (HIPAA) and the study IRB, but may be made available in a de-identified format following formal request to the corresponding author, which will be reviewed on a case-by-case basis.

## Abbreviations

SARS-CoV-2: Severe acute respiratory syndrome 2
ZAP COVID-19: Neutralizing Antibody Project for COVID-19
UCSD: University of California, San Diego
