# Supplementary material for: Longitudinal assessment of the impact of COVID-19 infection on mask-wearing behaviors
Source: BMC Public Health. 2024 Aug 16;24:2230. doi: 10.1186/s12889-024-19776-0 (PMC11328381; doi:10.1186/s12889-024-19776-0)
Supplement: Supplementary file 1 — Supplementary Material 1 [file 12889_2024_19776_MOESM1_ESM.docx]

| **Supplemental Table 1. Risk Perception and Masking Behaviors at Baseline by Patient Demographics** | | | | | | | | | | | | | |
| --- | --- | --- | --- | --- | --- | --- | --- | --- | --- | --- | --- | --- | --- |
|  | **Sex (n, %)** | | | **Race/Ethnicity (n, %)** | | | | | | | | **Age (mean, SD)** | |
|  | **Male** | **Female** | **P-val** | **NH White** | **Hispanic** | **Asian** | **Black** | **Am. Indian** | **PI** | **Other/**  **Mixed** | **P-val** | **Age** | **P-val** |
| **Risk Perception**  **Low**  **Average**  **High** | 178 (47.6)  136 (36.4)  60 (16.0) | 369 (44.3)  326 (39.1)  138 (16.6) | 0.40 | 275 (44.9)  235 (38.3)  103 (16.8) | 38 (37.6)  45 (44.6)  18 (17.8) | 141 (49.8)  98 (34.6)  44 (15.5) | 15 (57.7)  6 (23.1)  5 (19.2) | 2 (33.3)  3 (50.0)  1 (16.7) | 3 (30.0)  5 (50.0)  2 (20.0) | 54 (44.3)  47 (38.5)  21 (17.2) | 0.72 | 39.6 (14.1)  38.6 (12.3)  40.5 (11.1) | 0.06 |
| **Masking, outdoors**  **Never/Rarely**  **Sometimes**  **Usually/Always** | 80 (20.9)  67 (17.5)  236 (61.6) | 150 (18.1)  133 (16.0)  548 (65.9) | 0.14 | 149 (24.3)  106 (17.3)  359 (58.5) | 13 (13.0)  21 (21.0)  66 (66.0) | 26 (9.1)  39 (13.7)  220 (77.2) | 4 (14.8)  3 (11.1)  20 (74.1) | 2 (28.6)  1 (14.3)  4 (57.1) | 3 (30.0)  1 (10.0)  6 (60.0) | 22 (17.7)  22 (17.7)  80 (64.5) | <.001*** | 39.6 (12.0)  37.9 (12.1)  39.7 (13.5) | 0.05* |
| **Masking, indoors**  **Never/Rarely**  **Sometimes**  **Usually/Always** | 29 (7.6)  37 (9.7)  317 (82.8) | 46 (5.5)  63 (7.5)  728 (87.0) | 0.06 | 35 (5.7)  60 (9.7)  524 (84.7) | 6 (6.0)  7 (7.0)  87 (87.0) | 13 (4.5)  22 (7.7)  251 (87.8) | 1 (3.7)  1 (3.7)  25 (92.6) | 1 (14.3)  0 (0.0)  6 (85.7) | 2 (20.0)  3 (30.0)  5 (50.0) | 14 (11.3)  7 (5.6)  103 (83.1) | 0.05* | 42.5 (10.8)  38.3 (11.9)  39.4 (13.3) | 0.08 |
| **Masking,**  **public transport**  **Never/Rarely**  **Sometimes**  **Usually/Always** | 13 (5.9)  9 (4.1)  197 (90.0) | 22 (5.4)  19 (4.7)  367 (90.0) | 0.90 | 13 (4.6)  10 (3.5)  262 (91.9) | 1 (2.1)  1 (2.1)  45 (95.7) | 11 (6.3)  11 (6.3)  153 (87.4) | 0 (0.0)  0 (0.0)  12 (100.0) | 1 (25.0)  0 (0.0)  3 (75.0) | 1 (20.0)  0 (0.0)  4 (80.0) | 5 (7.1)  6 (8.6)  59 (84.3) | 0.17 | 38.9 (12.6)  34.0 (10.2)  36.3 (13.1) | 0.07 |
| **Masking,**  **other homes**  **Never/Rarely**  **Sometimes**  **Usually/Always** | 186 (56.0)  62 (18.7)  84 (25.3) | 415 (60.0)  133 (19.2)  144 (20.8) | 0.13 | 330 (64.5)  86 (16.8)  96 (18.8) | 57 (64.8)  13 (14.8)  18 (20.5) | 113 (46.9)  53 (22.0)  75 (31.1) | 12 (57.1)  3 (14.3)  6 (28.6) | 4 (57.1)  2 (28.6)  1 (14.3) | 4 (40.0)  3 (30.0)  3 (30.0) | 58 (54.7)  31 (29.2)  17 (16.0) | <.001*** | 38.1 (12.1)  41.3 (14.0)  39.7 (13.5) | 0.27 |

**Notes:** *p < 0.05; **p < 0.01; ***p < 0.001; unknown excluded from analysis for sex and race/ethnicity; Low risk includes responses: very low risk, low risk, and somewhat low risk; High risk includes responses: very high risk, high risk, and somewhat high risk
